# The statistical trade-off between word order and word structure – Large-scale evidence for the principle of least effort

**DOI:** 10.1371/journal.pone.0173614

**Published:** 2017-03-10

**Authors:** Alexander Koplenig, Peter Meyer, Sascha Wolfer, Carolin Müller-Spitzer

**Affiliations:** Institute for the German Language (IDS), Mannheim, Germany; University of Edinburgh, UNITED KINGDOM

## Abstract

Languages employ different strategies to transmit structural and grammatical information. While, for example, grammatical dependency relationships in sentences are mainly conveyed by the ordering of the words for languages like Mandarin Chinese, or Vietnamese, the word ordering is much less restricted for languages such as Inupiatun or Quechua, as these languages (also) use the internal structure of words (e.g. inflectional morphology) to mark grammatical relationships in a sentence. Based on a quantitative analysis of more than 1,500 unique translations of different books of the Bible in almost 1,200 different languages that are spoken as a native language by approximately 6 billion people (more than 80% of the world population), we present large-scale evidence for a statistical trade-off between the amount of information conveyed by the ordering of words and the amount of information conveyed by internal word structure: languages that rely more strongly on word order information tend to rely less on word structure information and vice versa. Or put differently, if less information is carried within the word, more information has to be spread among words in order to communicate successfully. In addition, we find that–despite differences in the way information is expressed–there is also evidence for a trade-off between different books of the biblical canon that recurs with little variation across languages: the more informative the word order of the book, the less informative its word structure and vice versa. We argue that this might suggest that, on the one hand, languages encode information in very different (but efficient) ways. On the other hand, content-related and stylistic features are statistically encoded in very similar ways.

## Introduction

Natural languages employ different strategies to transmit the information that is necessary to recover specific aspects of the corresponding message (e.g. grammatical relations, thematic roles, agreement, and more generally, the encoding of grammatical categories). While, for example, grammatical information ("who did what to whom") in a sentence is mainly conveyed by the ordering of the words in languages like Mandarin Chinese or Vietnamese, the word ordering is much less restricted for languages like Inupiatun or Quechua, as these languages (also) use the internal structure of words (e.g. the modification of word roots by inflection or the compounding of roots) as cues to inform about grammatical relationships in a sentence. This has led linguists to speculate, mostly qualitatively in nature [1–5], about a potential trade-off between the amount of regularity of the ordering of words and the amount of regularity of the internal word structure: languages that rely more on word order to encode information rely less on morphological information and vice versa. In this paper, we explicitly address this question quantitatively.

To this end, we apply one of the key ideas of the Minimum Description Length Principle: “any regularity in the data can be used to *compress* the data, i.e. to describe it using fewer symbols than needed to describe the data literally. The more regularities there are, the more the data can be compressed.” [6]. To illustrate this idea, let us turn to the English King James version of the Bible that consists of 5,070,889 characters (including spaces) in total (cf. Materials and methods for details on data processing and analysis). A very simple approach to compression would be the following: We first analyse the word-type-token distribution of this text in the spirit of Zipf [7] and count each occurrence of each distinct word (type) and rank the words according to their (token) frequencies; the most frequent word receives the first rank (*r* = 1), the second most frequent receives the second rank (*r* = 2), and so on. In a second step, we simply replace the first occurrence of each word type with its corresponding rank followed by an underscore and the actual word type, e.g. for the first occurrence of the second most frequent word type (“the”), we write “2_the”. For each further occurrence of a word type in the text, we only use its corresponding rank. This simple and crude procedure already compresses the original text to less than 70% of its original size (3,486,757 characters). It is worth pointing out that the original string is fully reconstructable from our “compressed” version. (*NB*.: A standard off-the-shelf file compressor is able to compress the original string to less than 25% of its original size by using more sophisticated compression methods than the one described above.) Our compression scheme works, because there are a few words that are repeated very often throughout the text. Those words receive shorter “codes” (in those cases: smaller integers). The rare word types that occur only once or twice necessarily receive longer codes (e.g. “12720_everlastingness”), but because the frequent word types tend to be so highly frequent, we are nevertheless able to compress the original string to a considerable extent. Interestingly, there is a direct link between this observation and the cognitive organisation of language as stipulated by Zipf [7]: on average, frequent words tend to be short; an observation that, according to Zipf, results from a need to communicate efficiently, something which is now know to be Zipf’s principle of least effort. While the observation that frequent words tend to be short holds across many or even all different natural languages [8], [9] provide empirical evidence that the amount of information conveyed by a word given its local context (i.e. a few words preceding a particular word token) is an even better predictor of word length than word frequency. Apart from providing insights into the cognitive organization of natural languages, this also demonstrates that statistical information plays a vital role on many different levels of linguistic structure. These different aspects of statistical information can then be used to compress natural language data.

In this paper, we are interested in measuring linguistic regularities both at the level of word structure and at the level of word order. As in [10], we refer to the former as intra-lexical regularity that manifests in the fact that “in many (or most) languages, words can be related to each other by (quasi-)regular relations between their orthographical forms, their grammatical functions, and their meanings.” In a similar vein, word order regularity can be defined as inter-lexical regularity where the relative order of words (partly) conveys important grammatical and semantic information. Relating this to the Minimum Description Length Principle, such regularities can be used to compress natural language data, because regularity translates into redundancy in the form of repetition throughout the text. Examples are repetitive word structure patterns, e.g. [VERB+ed] to express that an action started in the past, or recurrent word order constructions, e.g. putting the auxiliary verb before the subject to signal a question (“When **did** you leave?”).

Accordingly, our approach to empirically approximating the amount of redundancy at a specific text position *i* is based on the following idea: In order to determine the redundancy at position *i*, we examine the whole portion of the text up to (but not including) *i* and monitor how many of the initial characters of the text portion starting at *i* have already occurred in the same order somewhere in the preceding text, and record the length of longest continuous substring. Our key quantity of interest *l*_i_ is obtained by adding 1 to the longest match-length. As an example, imagine that we read the King James version of the Bible (here the Gospel of Matthew); let us assume that we have already read the first 127,348 characters of the text (again including spaces). Around the end of this text portion, the text reads “they perceiv**e**d that he spake of them”, where the letter e in boldface, i.e. the 13^th^ letter position of the sentence, is the final character read so far. At this position, we can go through the previous 127,347 characters and will find out that the longest contiguous subsequence starting at *i* and being a repetition of a sequence starting before this position can be found at position 125,150 (in boldface): “they suppos**ed that** they should have …”. Thus, at position *i*, the resulting sequence that approximates redundancy is “ed that”. Including spaces, that sequence is 8 characters long, so *l*_i_ = 9. Interestingly, [11] showed that *l*_i_ grows like (log *i*)/*H* where *H* is the entropy of the underlying process. Since *H* can be thought of as the “ultimate compression” of the string [12], *H* can be seen as a useful index of the amount of redundancy contained in the string (for convergence issues, cf. the Materials and methods section). However, as [13] demonstrate, *l*_i_ is highly dependent on the choice of *i*, e.g. it both fluctuates to a considerable extent and naturally depends on the amount of text that we have already read up to position *i*. To solve these problems, [13] simply suggest calculating *l*_i_ at each position *i* of the whole string with a length of *N* characters. The resulting estimates of redundancy at each position in the text are then averaged, which leads to the following estimator of the entropy of the string:

$$H=\;{\left[\frac{1}{N}\Sigma_{i=2}^{N}\frac{l_{i}}{\text{log}\left(i\right)}\right]}^{-1}$$ (1)

The intuitive idea behind this approach is that longer match-lengths are, on average, indicative of more redundancy in the text and, therefore, a lower mean uncertainty per character. (*NB*.: Formally, the correct mathematical notation would be to use the hat operator (“$\hat{H}$”) in this context since we only estimate the true entropy rate of the underlying process (“*H*”). For reasons of simplicity only, we do not use the hat operator throughout this paper, but explicitly note that by default, all entropy variables denote estimated values of their corresponding theoretical counterparts.)

Now, to isolate the amount of redundancy that can be attributed to the ordering of words, we first estimate the entropy of the original string based on (Eq 1) and call that quantity *H*_original_. We then randomize the word order, recalculate the entropy of the resulting string and call that value *H*_order_. The difference *D*_order_ between this entropy and the original estimate, *H*_order_−*H*_original_, then approximates the amount of redundancy that is contained in the ordering of words, with higher values being indicative of a greater amount of redundancy of the word order. Analogously, by estimating the entropy of a version of the string in which the intra-lexical regularities have been masked, by replacing all tokens for each word type with a unique equal-length sequence of characters randomly constructed from the characters available in the corresponding string. We then recalculate the entropy of that string and call that value *H*_structure_. Then, *D*_structure_ = *H*_structure_−*H*_original_ measures the amount of information that can be attributed to the word-internal structure, with higher values being indicative of a greater amount of redundancy of the word structure.

Theoretically, the trade-off hypothesis mentioned at the beginning of the introduction can be justified as another instantiation of Zipf’s principle of least effort, or the more general framework of synergetic linguistics [14]: If, for example, grammatical relationships in a sentence are fully determined by the ordering of words, it would constitute unnecessary cognitive effort to additionally encode this information with intra-lexical regularities. If, however, word ordering gives rise to some extent of grammatical ambiguity, we should expect this ambiguity to be cleared up with the help of word structure regularities in order to avoid unsuccessful transmission.

Without making any assumptions regarding the functional form of the relationship, we can compute Spearman's rank correlation coefficient *r*_s_ between *D*_order_ and *D*_structure_. A trade-off between both variables would imply that:

$$r_{s}\ll 0$$ (2)

From a mathematical point of view, the functional form of a trade-off hypothesis can be operationalized in terms of inverse proportionality: if we compare two different languages *m* and *n*, and assume that all other things are equal, if the value of *D*_*order*_ for language *m* compared to language *n* is greater by a certain magnitude, the value of *D*_*structure*_ should be lower by this magnitude, or that $D_{\text{order}}^{m}/D_{\text{order}}^{n}=D_{\text{structure}}^{n}/D_{\text{structure}}^{m}$. This implies that:

$$D_{\text{structure}}^{m}*\;D_{\text{order}}^{m}=\;D_{\text{structure}}^{n}*\;D_{\text{order}}^{n}$$ (3)

If we want this condition to hold for all possible pairs of languages, this implies that the product of both values, should always be the same for each language *m*:

$$D_{\text{structure}}^{m}*\;D_{\text{order}}^{m}\;=\beta$$ (4)

Since we estimate both quantities from available empirical data, we model *D*_order_ as a function of the reciprocal of *D*_structure_, where the conditional expectation can be written as:

$$E\left(D_{\text{structure}}|D_{\text{order}}\right)=\beta_{0}+\beta_{1}*{\left(D_{\text{order}}\right)}^{-1}$$ (5)

where the parameters *β*_0_ and *β*_1_ are estimated empirically.

In the Materials and Methods section, we present further technical details of our approach. In addition, it contains an extensive validation sub-section to support the credibility of our results.

## Results

**Fig 1:**
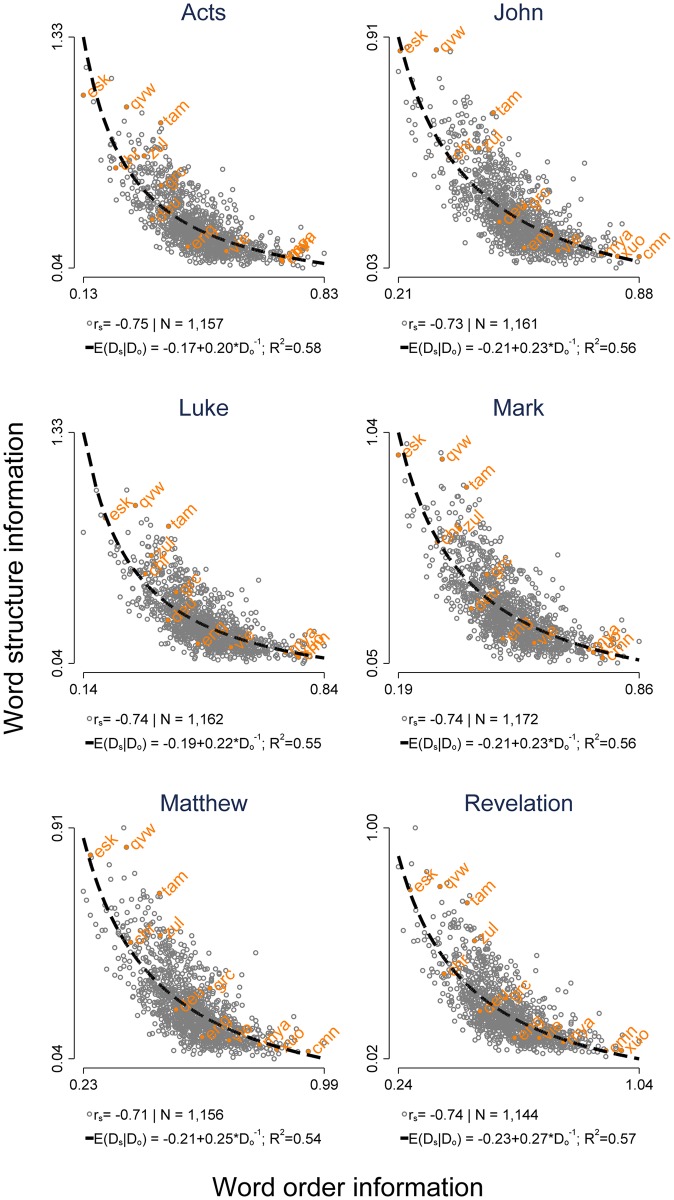
The statistical relationship between word structure information and word order information (in *bpc*). One plot for each of the six investigated books of the biblical canon. Orange labels show the ISO codes for twelve selected languages. *r*_*s*_-values are Spearman correlation coefficients. Black dashed lines in each plot indicate that a lot of structure can be captured in a simple model that suggests the presence of a reciprocal relationship (cf. Eq 5). Abbreviations: chr–Cherokee: cmn–Mandarin Chinese; deu–Standard German; eng–English; esk–Northwest Alaska Inupiatun; grc–Koine Greek; mya–Burmese; tam–Tamil; qvw—Huaylla Wanca Quechua; vie–Vietnamese; xuo–Kuo; zul–Zulu.

Fig 1 summarizes the primary result of our analysis. There is clear evidence for a statistical trade-off between the amount of word structure information and the amount of word order information. For all investigated books, there is a negative Spearman correlation of at least *r*_*s*_ = −.71.

The black dashed lines in Fig 1 show that a lot of the structure can be captured by a simple statistical model that suggests the presence of a reciprocal relationship (cf. Eq 5): variation in *D*_order_ explains at least 54% of the variation in *D*_structure_ for all six investigated books. Further results for all other books of the biblical canon can be found in the Materials and Methods section. In addition, interactive visualizations for all other books of the biblical canon are available online (cf. the Materials and methods section for further details). This relationship between word order information and word structure information corresponds well with typological expectations. Highly synthetic languages like Inupiatun (ISO code: esk) or Quechua (qvw) have a higher level of word structure information and a lower level of word order information, while very analytic languages like Mandarin Chinese (cmn), Vietnamese (vie) or Kuo (xuo; an Mbum language of southern Chad) primarily convey grammatical information by the ordering of words (among them grammatical particles that correspond to inflectional morphology in more synthetic languages). On the other side, very analytical languages show a high(er) level of word order information and a low(er) level of word structure information. Languages like Koine Greek (the original language of the New Testament, grc), German (deu), or English (eng) mix both methods of conveying information; accordingly, these languages tend to occupy intermediate spots on this spectrum.

**Fig 2:**
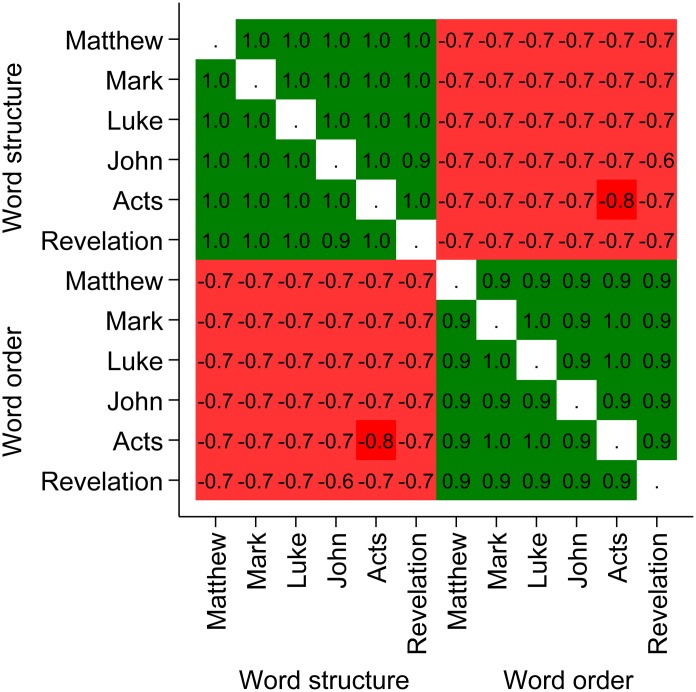
Spearman rank correlation matrix for all combinations of the six investigated books. The matrix visualizes that the word order and word structure information rankings have a strong positive intra-correlation and a strong negative inter-correlation.

To rule out the possibility of overfitting the data and to demonstrate the robustness of our results, Fig 2 shows that the resulting word order and word structure information rankings are strongly positively intra-correlated and strongly negatively inter-correlated. In terms of variance explained, if we know the word structure information ranking of Matthew, we can explain roughly *r*^2^ = .98^2^ = 96% of the variation in the word structure ranking in Acts and roughly *r*^2^ = −.74^2^ = 54% of the variation in its word order ranking.

Further validations of our approach can be found in the Materials and Methods section.

**Fig 3:**
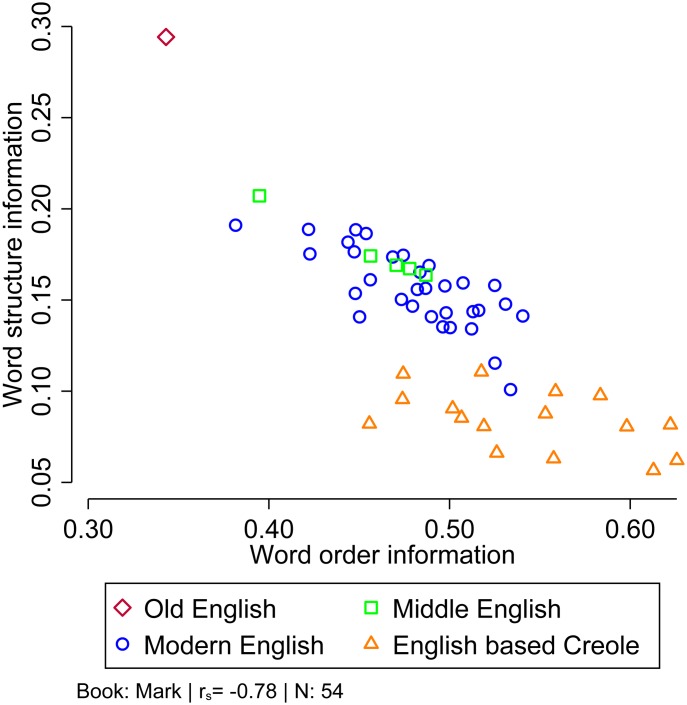
Relationship between word structure and word order for translations in English and English based Creole. Book: the Gospel of Mark.

Fig 3 shows that our results can also be interpreted from an evolutionary point of view. Here, we focus on Mark, since this is the only book for which we have textual data in Old English. Old English, typologically classified as a synthetic language, relies on morphological structure to convey grammatical information. Modern English uses analytic constructions to mark grammatical relations. This corresponds well with the visible trend in Fig 3, showing a substantial shift from Old English, with a high amount of word structure information, to both Middle and Modern English, where the system of inflectional endings was reduced in favor of a stricter word order as indicated by a higher amount of word order information: "The Middle English evolution consists primarily in a shift towards more analytic structure, eventually approaching that of today’s language which […] is close to isolating" [15].

For example, with the loss of inflections in the period of Late Old English / Early Middle English, it became difficult to identify a genitive case when an article or a possessive adjective was followed by a noun phrase. This ambiguity problem was solved by replacing genitives with *of*-constructions in the period of Middle English [16] and thus creating a higher amount of word order information. Or put differently, if less information is carried within the word, more information was spread among words in order to communicate successfully. For the evolution of languages, this indicates that a change in one grammatical area can trigger a temporally subsequent change in another grammatical area. This is exactly what [17] shows for Icelandic, where changes within its words lead to changes in its syntax.

The classification of the English based Creole languages is also interesting. Most prominently, McWhorter [18] argued that Creole grammars are relatively simply, particularly in terms of their morphology. Our results indicate that for English based Creoles, this is only one side of the story, as the substantial shift towards more analyticity with very little reliance on inflectional morphemes seems to be compensated by a strict word order to mark grammatical categories.

It is worth pointing out that Fig 3 could also be used as a validation of the two key quantities estimated in this paper: the amount of word order information and the amount of word structure information measure how a given language encodes information, in this case, regarding grammatical functions.

**Fig 4:**
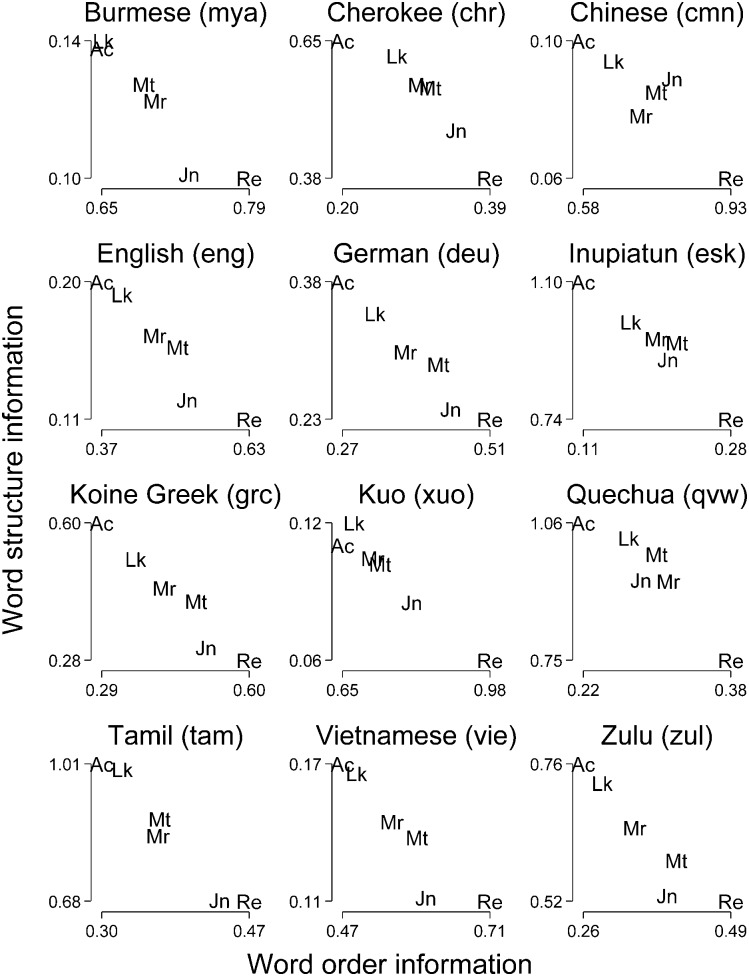
Inter-book trade-off for the six investigated books for twelve selected languages. Abbreviations: Ac–Acts; Jn–John; Lk–Luke; Mr–Mark; Mt–Matthew; Re–Revelation.

Remarkably, while Fig 2 clearly reveals that the inter-language word structure and word order rankings are strongly correlated, Fig 4 indicates that there also seems to be an inter-book trade-off in addition to the inter-language trade-off: the more informative the word order, the less informative the word structure and vice versa. If we calculate the Spearman correlation for the 6 investigated books, the median correlation for the 12 selected languages is *r*_*s*_ = −.94 (for all *N* = 1,476 translations, the median correlation is also *r*_*s*_ = −.94). If we compare the emerging pattern across languages, we find that the intra-language relationship is surprisingly similar: regardless of whether the languages are historically/geographically related or not, Revelation and John tend to have a higher word order information and a lower word structure information in relation to the other investigated books, and conversely for both Acts and Luke. Mark and Matthew tend to occupy intermediate positions. More precisely, if we calculate the correlations between all inter-book rankings for the selected languages, the median correlation is *r*_*s*_ = .94. For the word structure ranking, the lowest correlation is between Mandarin Chinese and Kuo with *r*_*s*_ = .71 (*p* = .068). For the word order ranking, the smallest correlation is between Cherokee and Quechua with *r*_*s*_ = .77 (*p* = .051). In sum, these results suggest that, while different languages occupy very different positions in the two-dimensional word order/word structure space, indicating differences in the way grammatical information is encoded, content-related and stylistic features of the source material (here: the Koine Greek version) are encoded in very similar ways when they are translated into different languages.

**Fig 5:**
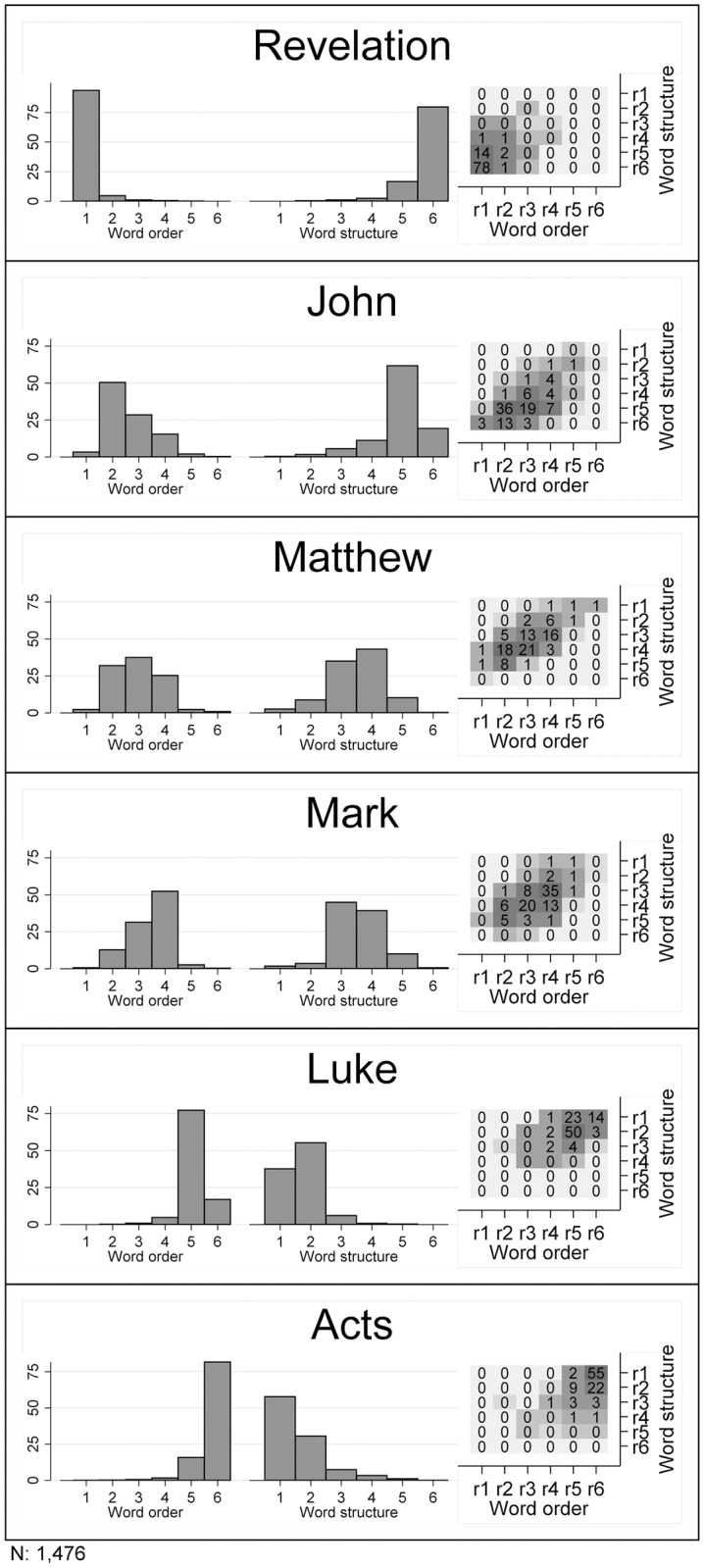
Word order information and word structure information rankings for the six investigated books. A rank of 1 indicates that the corresponding book has the highest word order or word structure information of all six books of the corresponding translation. Histograms visualizing the distribution of word order information ranks and word structure information ranks are depicted in the first two columns. The height of each bin represents the relative frequency of occurrence of the corresponding rank (in %). The matrix-plots (3rd columns) present bi-variate histograms, in which the colors of the cells represent the relative frequency of occurrence, with darker shades of gray representing a higher relative frequency (in %). In addition, numbers printed in each cell report relative frequencies rounded to the nearest integer.

To further explore this pattern in Fig 5, we used *N* = 1,476 translations for which we had available information for all six books. Here, we ranked the books, with a rank of 1 indicating that the corresponding book has the highest word order or word structure information of all six books for this translation. For each book, separate histograms visualizing the distribution of word order information ranks and word structure information ranks are depicted in the first two columns. The emerging inter-book pattern in Fig 5 is remarkably stable across translations: Revelation and John tend to have a higher word order information and a lower word structure information in relation to the other investigated books, while the opposite applies to both Acts and Luke. Mark and Matthew tend to occupy intermediate positions across translations. While most contemporary scholars do not believe that the Revelation of John and the Gospel of John were written by the same person, there is a widespread consensus that the Book of Acts and the Gospel of Luke were written by one author [19]. In general, metaphors, symbolism and the repetition of key phrases are characteristic for the Book of Revelation [20], while some scholars describe the author of Luke-Acts as a reliable historian accurately recording historic events and geographic places [21]. Regarding the four Gospels, there is also agreement about the fact that Mark, Matthew and Luke are distinct from John, both in content and in style, with John containing more metaphors or allegories [22].

Since our analysis has no liturgical goals, from a linguistic point of view, we conclude that one interpretation of this result is that stylistic and content-related properties of the source material seem to be preserved when translated into different languages. Or put differently, if a book of the biblical canon shows more word order regularity and less word structure regularity than another book in one language, then this relationship between the books is likely to reappear in other languages. More work is needed to understand the linguistic correlates of style and/or content that lead to the emergence of this pattern.

## Discussion

In this paper we used a simple quantitative information-theoretic approach that is not restricted to a particular language or a particular writing system. Moreover, the approach is not motivated by a specific linguistic theory and is less subjective than other more traditional ad-hoc measures [23]. However, it is important to point out that such a numerical approach also has drawbacks: (i) As it is completely based on the entropy estimation of symbolic sequences, the approach does not permit conclusions about specific word sequences (or alternative sequences) that can be used to encode a specific message. (ii) It does not reveal the precise underlying structures that are affected by the destruction of intra- and inter-word regularities [24]. (iii) Finding a definition for a word type that is valid across languages is harder than it might seem [25]. For example, since we destroy the intra-lexical structure of distinct space-separated sequences, word types that span two strings separated by a blank are excluded from this procedure. This can be problematic, since the orthographical representation of compounding varies across languages, (e.g. English "data set" vs. German "Datensatz"). Addressing these problems and developing additional validation methods are clearly required steps to assess the accuracy of our approach.

With that in mind, we hope that we were able demonstrate that our results can be interpreted in an intuitively plausible and meaningful linguistic way [26]. We presented evidence that supports Zipf's principle of least effort in relation to the way natural languages encode grammatical information. In addition, we found that–despite differences in the way information is encoded–the inter-book trade-off of different texts of the biblical canon regarding the amount of both word order information and word structure information remains highly similar across translations into different languages. On the methodological side, it arose as a by-product of our study, something we did not expect when we conducted this study, which indicates the great potential of quantitative studies both for gaining empirical evidence for long-standing claims and for finding out new aspects of language and its statistical structure. On the theoretical side, the result suggests that basic stylistic and content-related properties of individual texts are preserved when they are translated into different languages.

Conversely, the stability of the relationship across books that differ with regard to style and content strengthens the evidence for the statistical trade-off between word order and word structure. Overall, this is our major scientific finding: based on a quantitative analysis of more than 1,500 unique translations of different books of the Bible in almost 1,200 different languages, we found evidence for an inverse relation between the amount of information contributed by the ordering of words and the amount of information contributed by the internal word structure: languages that rely more on word order to transmit grammatical information, rely less on intra-lexical regularities and vice versa.

## Material and methods

We neither pursue liturgical or theological goals, nor do we want to propagate Christian missionary work. As linguists, our interest in the Bible stems solely from the fact that it is the book with the most available translations into different languages [27].

Raw data and code required to reproduce all results presented in this paper are available in Dataverse (doi:10.7910/DVN/8KH0GB). In addition, interactive visualizations (plus data and code) are available online at http://www.owid.de/plus/eebib2016/project.html.

As our data basis, we used the Parallel Bible Corpus made available by [27]. It contains 1,559 unique translations of the Bible in 1,196 different languages in a fine-grained parallel structure (regarding book, chapter and verse). Each translation is tokenized and Unicode normalized. Spaces were inserted between words and both punctuation marks and non-alphabetic symbols. In addition, all texts were manually checked and corrected by [27] where necessary. In texts without spaces or marks between words, a dictionary lookup method was used to detect word boundaries (e.g. for Khmer, Burmese, or Mandarin Chinese). Detected word tokens are space-separated. All uppercase characters were lowered based on the closest ISO 639–3 code. We then split each bible translation into different books of the biblical canon, effectively treating each book as a different text sample of the corresponding Bible translation. Here, we focused on the following six books of the New Testament: the four Gospels (Matthew, Mark, Luke, John), the Book of Acts and the Book of Revelation, because (i) we have sufficient available translations in different languages for those books and (ii) those books are reasonably long which makes the estimation of our two key quantities more reliable and robust. Interactive visualizations for all other books of the biblical canon are available online.

In October 2015, the Wycliffe Global Alliance estimated that almost 6 billion people have access to at least portions of the Bible in their native language [28]. To check the reliability of this figure, we extracted native speaker estimates for the languages available to us from the English Wikipedia [https://en.wikipedia.org, all accessed on 07/11/2016]. Our estimate corresponds well with the figure quoted above. It is important to emphasize that such a figure has to be treated with caution, because (i) census methods and dates of surveying vary significantly, (ii) defining a language, a language variety, or a dialect can be difficult, and (iii) there are people with more than one native language.

Let us represent each book as a symbolic sequence of *N* characters, i.e. *b* = {*c*_1_, *c*_2_, …, *c*_*N*−1_, *c*_*N*_} where *c*_*i*_ represents any character (including white spaces and punctuation marks) in the book at position *i*. The set of all distinct characters (or letters) that appear in *b* is defined as the alphabet *A*^b^, while the set of all distinct space-separated sequences (or word types) that appear in *b* is defined as the book’s lexicon *W*^b^. While this technical definition of word types is the de-facto standard in quantitative linguistics, this definition can be questioned from a theoretical point of view as mentioned above [25,29].

Since we are interested in measuring the amount of information that is conveyed by the word structure and the ordering of words, we use information theory as our mathematical framework [12]. We measure the entropy per symbol or entropy rate *H*^b^ for each book *b* which can be defined [12] as the average amount of information that is needed in order to describe *b*. Or put differently, *H*^b^ measures the redundancy of *b* [30]. We use the non-parametric estimation method of [13,30] that builds on the key idea of the Lempel-Ziv compression algorithm [11]. This method does not require any prior training, produces robust estimates without the need for very long strings as input and is able to take into account very long range correlations typical of literary texts [31,32] that are not captured by direct parametric Markovian or "plug-in" estimators [30]. For each book *b*, we estimate the per-symbol description length as ([30]; cf. Eq 1):

$$H^{b}=\;{\left[\frac{1}{N}\Sigma_{i=2}^{N}\frac{l_{i}}{\text{log}\left(i\right)}\right]}^{-1}$$ (6)

To measure the minimum number in bits per character [bpc], logarithms throughout this paper are taken to base two. Here, the key quantity of interest is the match-length *l*_*i*_. It measures the length of the shortest substring starting at position *i* of *b* that is *not* also a substring of the part of the book before this position. Alternatively, *l*_i_ can be obtained by adding 1 to the longest match-length as outlined in the Introduction and in [13]. There are no restrictions regarding the size of the "database", illustrating (i) why the estimator can be used in the presence of very long range correlations, as we do not impose any restrictions on how far "into the past we can look for a long match" [30] and (ii) that the estimator seems like a reasonable model of linguistic patterns of experience, as it captures structure on various levels of linguistic organization (co-occurring words, regular relations between grammatical word forms, constructions) that can be linked to theories of language learning and language processing [33]. Details of our Java program to efficiently obtain the match-length *l*_i_ at each position *i* for a given set of texts as well as an open source version can be found online.

It is important to note that the estimation of entropy rate is defined as the average description length for a process that is both *stationary* and *ergodic*. It is not clear *a priori* whether textual data can be considered such a process [12], or whether both concepts are even meaningful for natural languages [34]. To induce (at least some) stationarity, and thereby improve convergence, we randomized the order of the verses in each book, effectively discarding all supra-verse information [10].

Based on the ideas of [23,35,36], we approximate the amount of information that is conveyed by ordering of words and the structure of words, by estimating *H*^b^ for three versions of each book: (i) $H_{\text{original}}^{b}$ is estimated on the basis of the original version of the book. (ii) $H_{\text{order}}^{b}$ is estimated on the basis of a version of the book in which word ordering was deliberately destroyed. (iii) $H_{\text{structure}}^{b}$ is estimated on the basis of a version of the book in which intra-lexical regularities have been masked (see below for further explanation). Now, if we use the book version with absent word order to construct a code instead, we will need $H_{\text{original}}^{b}\;+\;D_{\text{order}}^{b}$ *bpc* on average in order to describe *b* where $D_{\text{order}}^{b}$ is the relative entropy [12,36]. Analogously, $D_{\text{structure}}^{b}=H_{\text{structure}}^{b}-H_{\text{original}}^{b}$. Thus, the incurring penalty of $D_{\text{order}}^{b}$ or $D_{\text{structure}}^{b}$ measures the amount of information in *bpc* that gets lost on average if the ordering of words or the intra-lexical structure is not considered when an efficient code is constructed in order to compress *b*. Hence, higher value of $D_{\text{order}}^{b}$ or $D_{\text{structure}}^{b}$ are indicative of a greater amount of regularity or information of the word order or the word structure.

Table 1 illustrates our approach of (i) destroying the word order and (ii) masking the word structure. For the version of the book with absent word order, we randomized the order of words within each verse. This means that when estimating the entropy rate of this book, redundancy that stems from the word order cannot be used to compress the corpus, but the statistics on the word level remains constant. In languages with a free relative ordering of words, this manipulation should not have a major influence. Hence, a small $D_{\text{order}}^{b}$ indicates that the relative ordering of words is less informative in the respective language. For the version of the book with masked word structure information, all tokens for each word type *w*_*j*_ ∈ *W*^*b*^ with a length of at least 2 characters are replaced by a unique equal-length sequence of characters randomly constructed from the alphabet *A*^b^, effectively destroying the structure on the word level, but keeping both the syntactical and the collocational structure constant. To illustrate our approach, line 2 of Table 1 shows that the masking procedure replaces each token of a word type by a unique equal-length random sequence of characters (eg. “called” is replaced by “itweiy” and “her” is replace by “khk”). Therefore intra-lexical regularities are being masked, e.g. the formation of the simple past tense via the suffix "ed" (*here*: “call**ed**” and “want**ed**”). Now, if the intra-lexical structure carries less information in a particular language, meaning that most words have little or no internal structure, this manipulation should not have a major influence on the entropy rate and thus lead to a small $D_{\text{structure}}^{b}$. Correspondingly, line 3 of Table 1 shows that when the word order is randomized, recurring word order patterns (*here*: “i called her”) cannot be used to compress the data anymore. In languages with a free relative ordering of words, this manipulation should not have a major influence on the entropy rate and thus lead to a small $D_{\text{order}}^{b}$.

**Table 1:** Toy example illustrating our approach.

Original	**i called her** yesterday and **i called her** today because **i** wanted **to** talk **to her**
Masked word structure	**i itweiy khk** doeerdsun rki **i itweiy khk** ehtuy ahuwlok **i** hwkilr **dw** weyy **dw khk**
Destroyed word order	**her** wanted **i** today talk **i i her** yesterday **called** because **called her to to** and

First line: original text. Second line: masked word structure. [*NB*.: "i" is not masked since it is only one character long. Thus it does not contain any intra-lexical structure.] Third line: destroyed word order. Word types printed in boldface appear at least two times.

It is worth pointing out that in both manipulated versions, basic quantitative structural properties of the original text remain unaffected (e.g. book length, word length, the type-token word frequency distribution), which rules out the (likely) possibility that changes to those characteristics influence the entropy rate estimation [37]. For the inter-book comparisons (cf. Figs 4 and 5), we keep *N* constant by first identifying the book with the smallest size in characters and then truncating the other five books at this position. In 1,450 of all 1,476 translations with available information for the six books, the shortest book is Revelation. Since we randomized the order of words within each verse for the book version with absent word order, differences of (average) verse lengths (in words) between the six books could potentially influence our results. To rule out this possibility, we generated an additional data set, where *N* is kept constant, but the word order is randomized per book instead of verse. The results based on this data set are qualitatively indistinguishable from the results we report here. The data set is also available online.

**Fig 6:**
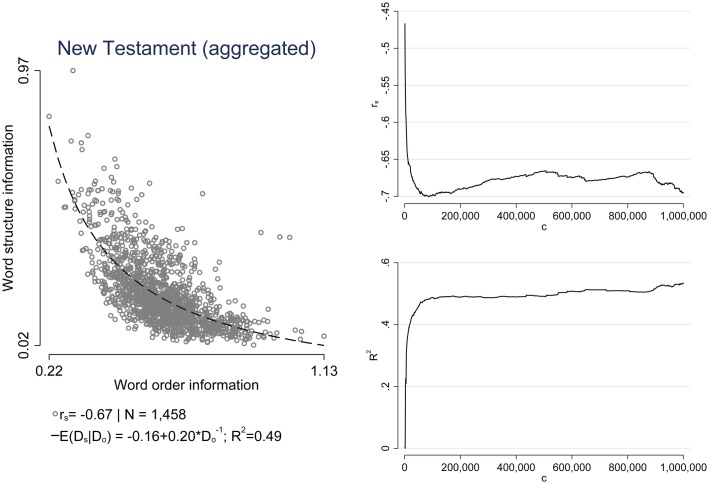
Aggregation of all books of the New Testament. Left side: Relationship between word structure information and word order information. Right side: Time series analysis of the data. *c* denotes the number of characters starting from the beginning of the text that we include in order to calculated *D*_order_ and *D*_structure_, so for example at *c* = 250,000, we include the first 250,000 characters of the string in one corresponding translation in order to calculate the relative entropies. Upper right side: Spearman correlation (*r*_s_) as a function of the amount of incorporated text (*c*). Lower right side: Coefficient of determination (*R*^2^) as a function of the amount of incorporated text (*c*). The results indicate that we do not need to include much data until a trade-off becomes apparent. [*NB*.: Only translations with available data for each New Testament book were included in the analysis. Both *D*_order_ and *D*_structure_ were not averaged across languages.]

To understand the functional form of the relationship between $D_{structure}^{b}$ and $D_{\text{order}}^{b}$ (cf. Eq 5 & Fig 1), we fitted the following non-linear regression function by least squares for each book *b*:

$$D_{\text{structure}}^{b,t}=\beta_{0}+\beta_{1}*{\left(D_{\text{order}}^{b,t}\right)}^{-1}+\epsilon^{b,t}$$ (7)

where *t* = 1, 2, …, *T* are our available translations for *b*; *ϵ*^*b*,*t*^ is the error term. For languages with more than one available translation $D_{\text{structure}}^{b}$ and $D_{\text{order}}^{b}$ were averaged across translations, except when stated otherwise in the text or in Figs 5 and 6, where we used all translations with available information for all six books.

*p*-values for the correlation of the rankings of the six books for the selected languages are based on exact permutation tests for all 6! = 720 permutations.

### Validation

In this subsection, we validate our quantitative approach with a focus on different potential problems in order to rule out the possibility that our results are only artifacts. To this end, we first compare our results with baseline data where spaces are first removed and then re-inserted randomly in order to show that the results are not a mere artifact of the way we define word boundaries. We then show that our approach stands a test for convergence and in a third step demonstrate that our results are not merely driven by varying string lengths.

It goes without saying that plausibility does not guarantee validity. Nevertheless, we think that it is worth emphasizing again that our main result corresponds well with linguistic typological expectations. So, from that point of view, our results are at least plausible. In this context, an important validation of our measure to approximate word structure regularity measure can be found in [38]. Here, the authors use human expert judgements using a varying number of features (1–27) from the World Atlas of Language Structures (WALS [39]) to rank different languages according to the amount of morphological structure. The resulting ranking is then compared to four quantitative measures automatically calculated from language corpora, one of those measures being *D*_structure_. While there are strong pairwise correlations between all four measures, of all four quantitative measures the ranking resulting from *D*_structure_ correlates best with the WALS ranking independent of number of included features (except for one of 27 cases, where the correlation between WALS and one different measure is slightly better). For example, if the WALS ranking is based on the maximum number of features, the Spearman correlation with *D*_structure_ reaches its maximum of .89.

#### Random insertion experiments

To allay concerns that our results might be artifacts of our approach, we proceed as follows: before the actual masking of the word structure and the randomization of the word order, we remove all spaces from the corpus data and then re-insert them into the strings in different but random ways in order to mask language-specific aspects of word boundary positions in the texts. At each step, we destroy more language-specific structure. Table 2 clearly demonstrates that the more language-specific structure we destroy the lower the statistical evidence for a trade-off between word structure and word order. The first column in Table 2 describes the corresponding experimental setting, while the second column presents an example of the resulting artificial text (the first 100 characters from the Book of Acts taken from the English King James Bible). Columns 3 to 14 present the corresponding Spearman correlation (*r*_s_) and the coefficients of determination of our parametric regression model (*R*^2^, cf. Eq 5) for each of the six investigated books. The number of cases in each simulation is 1,139 since we only included translations with available information for all six books. Let us now interpret the results:

(1) As a baseline we use the original string, since in the following simulations, spaces are randomly inserted into the string, verse borders are also destroyed. Thus, instead of randomizing the word order per verse, we now randomize the word order per book. The resulting trade-off correlations and model fits do not change strongly with this different randomization scheme, for all six investigated books, there is a negative Spearman correlation of at least *r*_*s*_ = −.70 and variation in *D*_order_ explains at least 52% of the variation in *D*_structure_.
(2) In order to check if the varying number of spaces between languages affects our results, we proceed with a first method test in which we did not actually remove and randomly re-insert spaces prior to the masking/randomization procedure, but only removed all spaces from the three versions of each book that were produced for baseline (1) prior to the estimation of match lengths. The results are even a little stronger than the original baseline with a negative Spearman correlation of at least *r*_*s*_ = −.73 and a model fit of a least *R*^2^ = .53. This indicates that the varying number of spaces does not influence the results.
(3) Here, we first remove all spaces from each book and then re-insert the spaces randomly, but keep the original word length distribution of the corresponding translation intact. After that manipulation, the three versions of the book (original, masked word structure, randomized word order) were prepared in the same way as for (1) and the resulting relative entropies were estimated. For all six books, the resulting negative Spearman correlation is not stronger than *r*_*s*_ = −.41 and *D*_order_ explains no more than 19% of the variation in *D*_structure_. These results are not surprising if we take into consideration that the word length distributions are very different for different languages. Consider, for example, Vietnamese (a language that relies more on word order, cf. Fig 1) with an average word length of *M* = 3.19 (*SD* = 1.56) and (Northwest Alaska) Inupiatun (a language that relies more on word structure) with an average word length of *M* = 9.08 (*SD* = 5.66). A language like German mixes both methods of conveying information according to our results; correspondingly its average word length occupies an intermediate spot on this spectrum (*M* = 4.26; *SD* = 2.55). Thus, when we re-insert spaces in a random but language-specific way, we should expect that the resulting trade-off would become much smaller compared to the original baseline, but remain existent. This is exactly what the simulation demonstrates.
(4) Again, we first remove all spaces from each book and then re-insert the spaces randomly. Compared to (3), we do not use the original word length distribution of the corresponding translation. We hypothesize that this should further reduce the Spearman correlations and the model fits. Nevertheless, we still expect to see some evidence for a trade-off relationship, because the number of spaces, which is equal to the number of words (plus one), is also language-specific. Another look at the three language examples confirms this: Vietnamese– 29,183 words, German– 26,582 words and Inupiatun– 14,823 words. Again, the results of our experiment validate our expectation: For all six books, the resulting negative Spearman correlation is not stronger than *r*_*s*_ = −.28 and *D*_order_ explains no more than 9% of the variation in *D*_structure_.
(5–7) For these three simulations, we again first remove all spaces from each book. Here, however, the number of spaces that we randomly re-insert is calculated based on the number of words across all translations for the corresponding book. For (5), we use the median number of words. For (6), we use the lower quartile (p25) of words that on average leads to longer “words”. For (7), we use the upper quartile (p75) of words that on average leads to shorter “words”. Since we destroyed even more linguistic information for those three simulations in comparison to (4), we expect the trade-off relationship to vanish completely. Again, the results of our experiment confirm this expectation: for all six books, the resulting negative Spearman correlation is not stronger than *r*_*s*_ = −.14 and *D*_order_ explains no more than 5% of the variation in *D*_structure_ in any of the three simulations. In some cases, there are even positive Spearman correlations instead of negative ones.
(8) As a final method test, we remove all word order and word structure information from the data in order to test the sensitivity of our approach. This is accomplished by masking the word structure and randomizing the word order. The only remaining information can be attributed to the Zipfian type-token distribution of words. Of course, our expectation in this case would be that there is no correlation at all if we run our approach with this artificial data a second time. The results clearly confirm this, there is no negative Spearman correlation for any of the six investigated books and our regression model no longer explains anything.

**Table 2:** Random insertion experiments.

Description	text_example	Ac		Jn		Lc		Mr		Mt		Re	
		*r*_s_	*R*^2^	*r*_s_	*R*^2^	*r*_s_	*R*^2^	*r*_s_	*R*^2^	*r*_s_	*R*^2^	*r*_s_	*R*^2^
1 Original string	and suddenly there came a sound from heaven as of a rushing mighty wind, and it filled all the hous	-0.75	0.57	-0.72	0.55	-0.73	0.55	-0.73	0.55	-0.70	0.52	-0.73	0.55
2 String without spaces	andsuddenlytherecameasoundfromheavenasofarushingmightywind,anditfilledallthehousewheretheyweresittin	-0.75	0.54	-0.73	0.53	-0.74	0.53	-0.74	0.53	-0.73	0.53	-0.75	0.54
3 Space insertion: language specific word length	andsudde nly t her ec ameasound fromh eaven asof a r us hing might ywi nd,an ditf illedal lthe house	-0.32	0.10	-0.41	0.19	-0.32	0.11	-0.32	0.11	-0.34	0.13	-0.30	0.11
4 Space insertion: language specific no.	and s udd e nl ytherec ame asoundf r omheave n asofar ushingmightywi nd,a nditf ill edallthe housew	-0.16	0.03	-0.28	0.09	-0.15	0.03	-0.17	0.04	-0.18	0.04	-0.19	0.05
5 Space insertion: no. according to p50 (median length words)	a nds uddenl ythere c amea s oundfromheavena sofa r ushin gmig htywi nd, andit fil led allthehousewh	0.13	0.00	-0.05	0.01	0.08	0.00	0.10	0.00	-0.02	0.01	-0.08	0.03
6 Space insertion: no. according to p25 (longer words)	an ds uddenly therec ameas oundfromheavena sofa ru shing mi g htywi nd, andit filled allthehousew he	0.09	0.00	-0.09	0.02	0.03	0.00	0.06	0.00	-0.08	0.02	-0.14	0.05
7 Space insertion: no. according to p75 (shorter words)	an ds uddenly th ere came a soundfromheavenas ofa r ushin gmig htywin d,a ndit fil led allthehousew	0.18	0.01	-0.01	0.01	0.13	0.01	0.14	0.01	0.04	0.00	-0.02	0.01
8 Method test: randomization/masking applied twice	?) d aj?;t jwyamx)v- logn hf p?s bg:fb. (wsf(?ikw v'j.pc (?fkjso a‴.k v'j i z?s mi bui -ier nr: s	0.08	0.00	0.01	0.00	0.07	0.00	0.03	0.00	0.07	0.00	0.06	0.00

For these experiments, all spaces were removed from the corpus data and then re-inserted into the strings in different but random ways prior to the actual masking of the word structure and the randomization of the word order. At each step, we destroy more language-specific structure. The more language-specific structure we destroy the lower the statistical evidence for a trade-off between word structure and word order. 1st column: description of the corresponding experimental setting. 2nd column: example of the resulting artificial text (the first 100 characters from the Book of Acts taken from the English King James Bible). 3rd– 14th columns: corresponding Spearman correlation (*r*_s_) and coefficients of determination (*R*^2^, cf. Eq 5).

Overall, we believe that the tests presented in this section strengthen the confidence in our quantitative approach: the more language-specific structure we destroy the lower the statistical evidence for a trade-off between word structure and word order.

#### Convergences issues

While [13] demonstrate that the entropy estimator used in this paper seems to converge to the source entropy very quickly, the entropy rate, as written above, is defined as the average description length for a process that is both *stationary* and *ergodic*. If we ask the question whether natural language data can be seen as such a process, we could put it as [12]: “Probably not!”. Both the study of [30] and the study of [40] point towards the fact that natural languages are nonergodic. Our results (cf. Figs 4 and 5) could be taken as one potential explanation why this is the case. Maybe even more importantly, pointwise convergence to the source entropy is only guaranteed in the limit [41], i.e. when the text size approaches infinity, something which is clearly not given for any linguistic corpus data whatsoever. Thus, it is essential to ask whether (i) our results depend on varying string lengths between different languages and (ii) whether our entropy estimates seem to converge.

Tables 3 and 4 summarize our attempts to answer those questions. The first column in Table 3 describes the corresponding experiment, while columns 2–19 present the corresponding Spearman correlation (*r*_s_), the coefficients of determination of our regression model (*R*^2^, cf. Eq 5) and the number of used cases (*N*) for each of the six investigated books.

(i) To determine whether our results change if we keep the string size per book across languages constant, we first discarded the lower 10% of translations per book with regard to string size (in characters), because some of the translations for a given book in the Parallel Bible Corpus are incomplete. For the remaining 90% of translations for a given book, we kept the string size constant by truncating all translations at the size of the shortest translation. As can be seen in the third row of Table 3, the resulting trade-off correlations and model fits strongly indicate that the results are not an artifact of varying string sizes across languages; for all six investigated books, there is a negative Spearman correlation of at least *r*_*s*_ = −.72 and variation in *D*_order_ explains at least 55% of the variation in *D*_structure_.
(ii) To check for convergence, we used a generic version of the convergence test by [36]. To this end, we calculated our three entropy estimates on the basis of the first 50% of the characters of each translation and each corresponding book. We then compared the resulting estimates with the estimates based on the full books. Only cases with a maximum discrepancy of 10% for any of the three entropy estimates (*H*_original_, *H*_structure_, *H*_order_) were used for the further analysis. The fourth row of Table 3 demonstrates that the results remain stable: for all six investigated books, there is a negative Spearman correlation of at least *r*_*s*_ = −.70 and variation in *D*_order_ explains at least 50% of the variation in *D*_structure_. We then re-ran this test with two different settings: in (Eq 3) we computed the entropies based on the first 75% of the texts and used a maximum discrepancy of 5% as a test criterion. In (Eq 4) we computed the entropies based on the first 87.5% and discarded all cases with discrepancy of more than 2.5%. In both scenarios, the results remain stable: for all six investigated books, there is a negative Spearman correlation of at least *r*_*s*_ = −.69 and variation in *D*_order_ explains at least 50% of the variation in *D*_structure_.

**Table 3:** Further validation tests.

Description	Ac			Jn			Lk			Mr			Mt			Re		
	*r*_s_	*R*^2^	*N*	*r*_s_	*R*^2^	*N*	*r*_s_	*R*^2^	*N*	*r*_s_	*R*^2^	*N*	*r*_s_	*R*^2^	*N*	*r*_s_	*R*^2^	*N*
1 Constant string size across languages	-0.75	0.57	1089	-0.75	0.57	1090	-0.74	0.55	1091	-0.74	0.56	1100	-0.72	0.55	1089	-0.74	0.56	1080
2 Convergence test: Entropies computed for first 50% of the string. Test criterion: maximum discrepancy of 10%	-0.73	0.52	1,497	-0.70	0.51	1,486	-0.72	0.50	1,495	-0.71	0.50	1,490	-0.70	0.51	1,135	-0.73	0.53	1,159
3 Convergence test: Entropies computed for first 75% of the string. Test criterion: maximum discrepancy of 5%	-0.73	0.53	1,498	-0.70	0.51	1,493	-0.72	0.50	1,503	-0.71	0.50	1,505	-0.70	0.51	1,302	-0.73	0.54	1,313
4 Convergence test: Entropies computed for first 87.5% of the string. Test criterion: maximum discrepancy of 2.5%	-0.73	0.53	1,493	-0.69	0.51	1,481	-0.72	0.50	1,494	-0.71	0.50	1,478	-0.69	0.51	1,276	-0.73	0.54	1,283

First column: description of the corresponding experiment. 2nd– 19th columns: corresponding Spearman correlation (*r*_s_), coefficients of determination of our regression model (*R*^2^, cf. Eq 5) and the number of used cases (*N*) for each of the six investigated books. The different experiments demonstrate that our results remain stable if we control for string length (3rd row) and test for convergence (4th to 6th rows).

**Table 4:** Spearman correlation for all Bible books with more than 100 available translations.

Book	*N*	p50	p25	p75	*r*_s_
New Testament (aggr.)	1,458	1,190,346	1,023,099	1,415,885	-0.67
Psalms	289	235,757	215,049	258,810	-0.64
Jeremiah	265	227,111	208,214	245,870	-0.60
Ezekiel	263	207,793	187,347	223,951	-0.57
Genesis	329	202,215	187,136	219,333	-0.67
Isaiah	268	200,312	180,181	217,992	-0.69
Exodus	288	167,778	152,407	179,644	-0.56
Numbers	273	166,516	151,811	178,742	-0.54
Luke	1,510	158,790	138,673	186,416	-0.72
Acts	1,503	154,551	135,619	181,419	-0.73
Matthew	1,505	149,968	129,413	177,136	-0.69
Deuteronomy	274	145,874	132,937	155,173	-0.63
2 Chronicles	262	141,210	129,966	151,156	-0.67
1 Samuel	273	131,388	121,394	140,917	-0.65
1 Kings	270	128,621	119,512	138,793	-0.55
Leviticus	268	124,535	113,310	133,107	-0.63
2 Kings	271	123,436	113,789	133,280	-0.55
John	1,510	117,368	103,763	136,171	-0.70
1 Chronicles	262	112,571	104,484	121,301	-0.70
2 Samuel	271	108,387	100,484	116,970	-0.67
Judges	270	99,302	91,652	106,156	-0.63
Job	266	98,125	90,281	106,023	-0.63
Joshua	278	97,839	89,204	104,940	-0.70
Mark	1,522	92,298	81,313	107,365	-0.71
Proverbs	274	83,315	75,247	91,593	-0.72
Revelation	1,484	74,298	63,806	87,312	-0.73
Romans	1,490	69,143	57,028	83,929	-0.73
1 Corinthians	1,490	66,713	55,221	81,014	-0.73
Daniel	266	63,959	58,564	69,546	-0.57
Nehemiah	267	57,808	53,455	62,972	-0.73
Hebrews	1,486	49,681	41,454	62,414	-0.74
2 Corinthians	1,487	43,044	36,452	52,622	-0.72
Ezra	265	39,973	36,356	42,986	-0.72
Zechariah	263	33,669	30,521	35,929	-0.65
Esther	266	30,716	28,491	33,584	-0.64
Ecclesiastes	266	29,766	27,505	32,373	-0.62
Hosea	265	29,030	26,311	32,349	-0.69
Galatians	1,490	23,290	19,076	28,949	-0.71
Amos	263	23,084	20,648	25,051	-0.54
Ephesians	1,490	21,908	18,335	26,879	-0.75
Lamentations	261	18,641	16,958	20,584	-0.71
1 Peter	1,484	18,144	15,024	22,161	-0.75
1 Timothy	1,495	18,056	14,851	21,850	-0.75
Micah	266	16,858	15,231	18,507	-0.68
James	1,495	16,495	13,699	20,082	-0.72
1 John	1,493	16,199	13,881	19,220	-0.74
Philippians	1,489	15,623	13,201	18,593	-0.70
Colossians	1,487	15,024	12,421	18,207	-0.74
Song of Solomon	257	14,857	13,827	16,387	-0.56
Ruth	299	13,534	12,558	14,710	-0.68
1 Thessalonians	1,495	13,183	11,197	15,665	-0.71
2 Timothy	1,489	12,546	10,409	15,092	-0.70
2 Peter	1,485	11,271	9,516	13,665	-0.73
Joel	265	10,943	9,987	11,743	-0.68
Malachi	265	10,115	9,297	11,277	-0.57
Zephaniah	260	8,902	8,038	9,773	-0.72
Habakkuk	262	8,248	7,523	9,137	-0.51
Titus	1,496	7,628	6,221	9,356	-0.75
2 Thessalonians	1,496	7,157	6,199	8,560	-0.70
Nahum	262	7,121	6,417	7,851	-0.62
Jonah	298	7,021	6,502	7,768	-0.64
Haggai	262	6,142	5,624	6,614	-0.67
Jude	1,479	5,064	4,126	6,306	-0.67
Obadiah	259	3,485	3,180	3,818	-0.69
Philemon	1,484	3,183	2,687	3,778	-0.65
3 John	1,481	2,183	1,827	2,587	-0.59
2 John	1,481	1,974	1,700	2,317	-0.61

1st column: name of the corresponding book. 2nd column: number of available translations. 3rd– 5th columns: median (p50), lower quartile (p25) and upper quartile (p75) of the string length per translation. 6th column: Spearman correlations (*r*_s_). [*NB*.: the data are sorted in descending order according to the median string length per book. For the calculation of the Spearman correlations, *D*_order_ and *D*_structure_ were not averaged across languages.]

It is important to point out that this test does not prove that we can be sure that convergence has been achieved in a strict sense. Still, the entropy rate of any process is defined in the limit, i.e. for infinitely long text segments. In conjunction with the study of [13], wo demonstrate that convergence for the estimator used in this paper is very fast when applied to natural language data, we believe that our results are good estimates of the corresponding source entropies.

To further demonstrate that our results do not depend on varying string lengths, Table 4 presents results for all Bible books with more than 100 translations. In addition, we aggregated all books of the New Testament to one large string (here, only translations with available data for each New Testament book were included in the analysis). For each book, we then calculated Spearman correlations (*r*_s_; last column in Table 4). The third, fourth and fifth column add information regarding the median (p50), lower quartile (p25) and upper quartile (p75) of the string lengths across languages for each corresponding book. The results are sorted by the median string length in descending order. As can be seen, there is a clear tendency of a strong trade-off between word order and word structure independent of the length of the corresponding book.

Since we are interested in the amount of redundancy that can be attributed to either the word structure or the word order and a potential trade-off between the two quantities, we conducted one additional analysis. Here, we mapped each available aggregated New Testament “translation” onto a time series $\left\{s\left(c\right)\right\}{\;}_{c=1}^{1,000,000}$ where *c* is the number of characters starting from the beginning of the text that we include in order to calculate *D*_order_ and *D*_structure_. For example at *c* = 250,000, we include the first 250,000 characters of the corresponding string to calculate the relative entropies. We then merged the available time series of each translation and calculated both the Spearman correlation (*r*_s_) and the model fit of our regression model (*R*^2^) at each position *c* starting at *c* = 1,000. As can be seen from the two plots on the right hand side of Fig 6, we do not need to include much data until a trade-off becomes apparent: at around 100,000 included characters our quantities of interest (*r*_s_ and *R*^2^) become stable.

Taken together, we hope that the validations presented in this section further strengthen the evidence for the statistical trade-off between word order and word structure.

**Fig 7:**
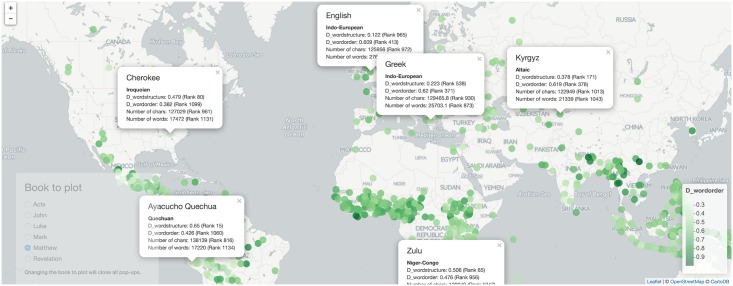
Screenshot illustrating the use of our interactive analysis tool that is available online.

The reader is invited to further explore our results interactively at http://www.owid.de/plus/eebib2016/project.html. For example, Fig 7 presents a screenshot of an interactive map. It reveals a typical pattern of linguistic features, i.e. "a strong tendency to geographical homogeneity" [2].
